# Retraction Note: Treatment of temporomandibular joint internal derangement using MESNA injection

**DOI:** 10.1186/s12903-025-06564-4

**Published:** 2025-07-14

**Authors:** Ahmed A. Mosleh

**Affiliations:** Oral and Maxillofacial Surgery, Faculty of Dentistry, Tanta University, Tanta, Egypt

**Retraction Note: BMC Oral Health (2024) 24:894**

10.1186/s12903-024-04615-w

The Editor has retracted this article. Concerns were raised over discrepancies between the data in the study and that in the clinical registry as regards the timeline, enrollment, and follow-up period of the actual research. For example, the record shows the study as completed on 1 April 2024 with the last update on 10 June 2024 - however, the paper was submitted on 22 March 2024. Additionally, the ethics approval seems to be dated by April 2023 and the study seems to have commenced in May 2023; however if this is the case, the submission in March 2024 contradicts the article’s statement that it contains data from one-year postoperative follow-up. The authors did not respond to a request to clarify these discrepancies. The authors did not respond to correspondence from the Editor about this retraction.

